# Financial development and environmental quality in developed countries: a systematic literature review

**DOI:** 10.1007/s11356-023-30557-x

**Published:** 2023-11-03

**Authors:** Ambepitiya Wijethunga Gamage Champa Nilanthi Wijethunga, Mohammad Mafizur Rahman, Tapan Sarker

**Affiliations:** 1 School of Business, University of Southern Queensland, West Street, Toowoomba, QLD 4350 Australia; 2 Department of Accountancy & Finance, Faculty of Management Studies, Sabaragamuwa University of Sri Lanka, Belihuloya, 70140 Sri Lanka; 3 School of Business, University of Southern Queensland, Springfield Education City, 37 Sinnathamby Blvd, Springfield Central, Ipswich, QLD 4300 Australia

**Keywords:** Financial development, Environmental quality, Carbon emissions, Developed countries, Systematic literature review

## Abstract

Studying the effect of financial development on environmental quality has become imperative in the modern world due to the climate change challenges. Hence, this systematic literature review provides a comprehensive overview of the existing body of knowledge on the nexus of financial development and environmental quality in developed countries. Three databases: Web of Science, Scopus, and Google Scholar were used to search the relevant articles in this domain. Finally, 20 journal articles qualified for the systematic literature review based on the pre-defined article inclusion criteria as per the Preferred Reporting Items for Systematic Reviews and Meta-analyses (PRISMA) framework. We found that a range of econometric approaches were used in all examined papers, employing a diverse range of proxy variables to model the relationship between financial development and environmental quality. Overall, the findings of the examined papers imply mixed evidence of this nexus in developed countries. We highlight the knowledge gap in this research domain examining the financial development and environmental quality link from different proxies.

## Introduction

In the present-day context, human activities have been altered and impair the dynamic stability of the ecological system, ultimately contributing to climate change (Debone et al. 2021). The detrimental waves of climate change directly impact human life, well-being, and well-being of future generations (Zafar et al. 2021). Thus, international associations such as United Nations Environmental Program, Intergovernmental Panel on Climate Change (IPCC), and others have prioritized efforts targeted at mitigating the adverse impacts of environmental variability, with steps to reduce carbon emissions (Acheampong et al. 2019; Tamazian et al. 2009) and various commitments have been made by countries to prioritize environmental protection as a key agenda in their policies (Ruza and Caro-Carretero 2022). However, to effectively implement these agendas, support from the financial systems within the economy is vital because the transition process towards a carbon-neutral economy would not be feasible without sufficient financing options to facilitate the necessary changes in the economic model and promote the transition of highly polluting sectors (Ruza and Caro-Carretero 2022).

In addition, the advancement of financial institutions and financial markets is a dominant aspect of economic progress that enhances the economic efficiency of the financial system in the economy (Hafeez et al. 2019). Financial development brings numerous economic benefits for both individuals and the corporate sector. It permits households and the corporate sector to access capital and credit at the lowest cost, thereby generating wealth which ultimately leads to boosting economic progress (Acheampong 2019). It assists in investing in green energy and energy-incentive technologies aiming for environmental sustainability (Dada et al. 2022). Moreover, financial development overcomes the barriers to investors (Acheampong 2019; Hafeez et al. 2019; Tamazian et al. 2009). On the other hand, financial development is challenging environmental quality in numerous ways. Financial development relaxes the barriers to investors by facilitating capital which promotes new capital investments; ultimately consumes more energy resources that emit more greenhouse gasses into the environment (Baloch et al. 2019; Saud et al. 2018; Hafeez et al. 2019). Similarly, weaker financial development could facilitate to channel financial resources to non-environmental friendly economic activities (Pata 2018).

Owing to its significance, a growing number of scholarly works have been dedicated to analyzing the link between financial development and environmental quality in different economic settings (Aljadani 2022; Hunjra et al. 2020; H. Khan et al. 2022; Konuk et al. 2023; Musah et al. 2022; Shahbaz et al. 2016; Vo et al. 2021). To this end, various scholarly works have modeled the financial development and environmental quality by adopting different econometric techniques and proxies under diverse time lengths in both developed and developing country contexts. More importantly, developed economies have reached tremendous development in financial markets and financial institutions (Zeeshan et al. 2021). However, the precise extent of its impact on environmental quality in developed countries’ context remains uncertain because both financial development and environmental quality are intricate and multifaceted concepts, including various dimensions, indicators, and variables, as well as the varying dynamics observed in different developed economies. Accordingly, this study addresses a fundamental research question: What is the existing knowledge wisdom regarding the relationship between financial development and environmental quality in developed economies?. By conducting an in-depth review of the existing empirical studies pertaining to the relationship between financial development and environmental quality, we seek to shed light on this relationship and provide a clear view of how financial development influences environmental quality in the context of developed economies.

To our knowledge, no study synthesizes the existing body of knowledge on financial development and environmental quality in developed countries. Thus, this Systematic Literature Review (SLR) aims to explore the common consensus of the existing body of knowledge regarding the relationship between financial development and environmental quality in the developed country context by synthesizing existing empirical works. It also assesses the adequacy of the common understanding of financial development and environmental quality to generalize the possibility of using the knowledge in making sensible policy decisions. Furthermore, the study attempts to discover the existing knowledge gaps in the study field for future research purposes. It will assist the researchers in advancing to investigate the impact by adding financial development and environmental quality proxies that were missed in the existing literature in developed countries.

This SLR adheres to the recommended selection guideline of the Preferred Reporting Items for Systematic Reviews and Meta-analyses (PRISMA) statement, whereas empirical studies on financial development and environmental quality appear to have grown over the years. Hence, it is crucial to comprehensively understand and synthesize the available data to minimize the risk of overlooking relevant information during the SLR. Thus, we have limited the articles published up to the 1st of June 2023 on Web of Science, Scopus, and Google Scholar to mitigate the risk. Furthermore, this study analyzed a total of 20 articles out of the initial selection of 277 studies, from developed-single country studies that met the inclusion and exclusion criteria of this SLR to understand the impact of financial development on environmental quality in developed economies. The rest of this SLR is structured as follows: “Research methodology” provides a detailed discussion of methods adopted in this study for searching the existing literature, article inclusion and exclusion criteria, data extraction and summarization, and quality assessment of the eligible papers to SLR. “Study sample credentials” discusses the study sample credentials. “Results” summarizes the major study characteristics and key findings of examined papers of the SLR. “Discussion” discusses the key findings of the study. Finally, “Conclusion” concludes the paper.

## Research methodology

This study utilized the Preferred Reporting Items for Systematic Reviews and Meta-analyses (PRISMA) statement, which was developed by Moher et al. (2009), as a framework for systematically presenting the existing body of literature on financial development and environmental quality link in a developed country context. The PRISMA statement facilitates scholars in producing concise and transparent literature reviews in different academic disciplines (Liberati et al. 2009; Mardani et al. 2019). Identification of existing literature, assessment of eligibility of existing literature to the SLR, information extraction from eligible articles, and synthesis of findings into a concise summary are the key steps in the PRISMA framework (Alkhars et al. 2022; Mardani et al. 2019). Additionally, studies by Consedine et al. (2015), Debone et al. (2021), Mardani et al. (2019), and Zubair et al. (2023) have employed the PRISMA framework to systematically conduct literature reviews in various disciplines.

### Searching the extant literature

In the initial phase, an extensive literature search was conducted across three databases, namely (1) Scopus, (2) Web of Science, and (3) Google Scholar, in order to detect the extant literature pertaining to financial development and environmental quality. The search strings for each database and the number of non-exclusive results that were generated are shown in Table 1.

**Table 1:** Extant literature search strings for databases

Keywords	Number of results (non-exclusive)		
	Scopus	Web of Science	Google Scholar
“Financial Development” AND “Environmental Quality”	94	64	109
“Financial Development” AND “Environmental Degradation”	91	84	49
“Financial Development” AND “Carbon Emission”	51	137	109

**Fig. 1:**
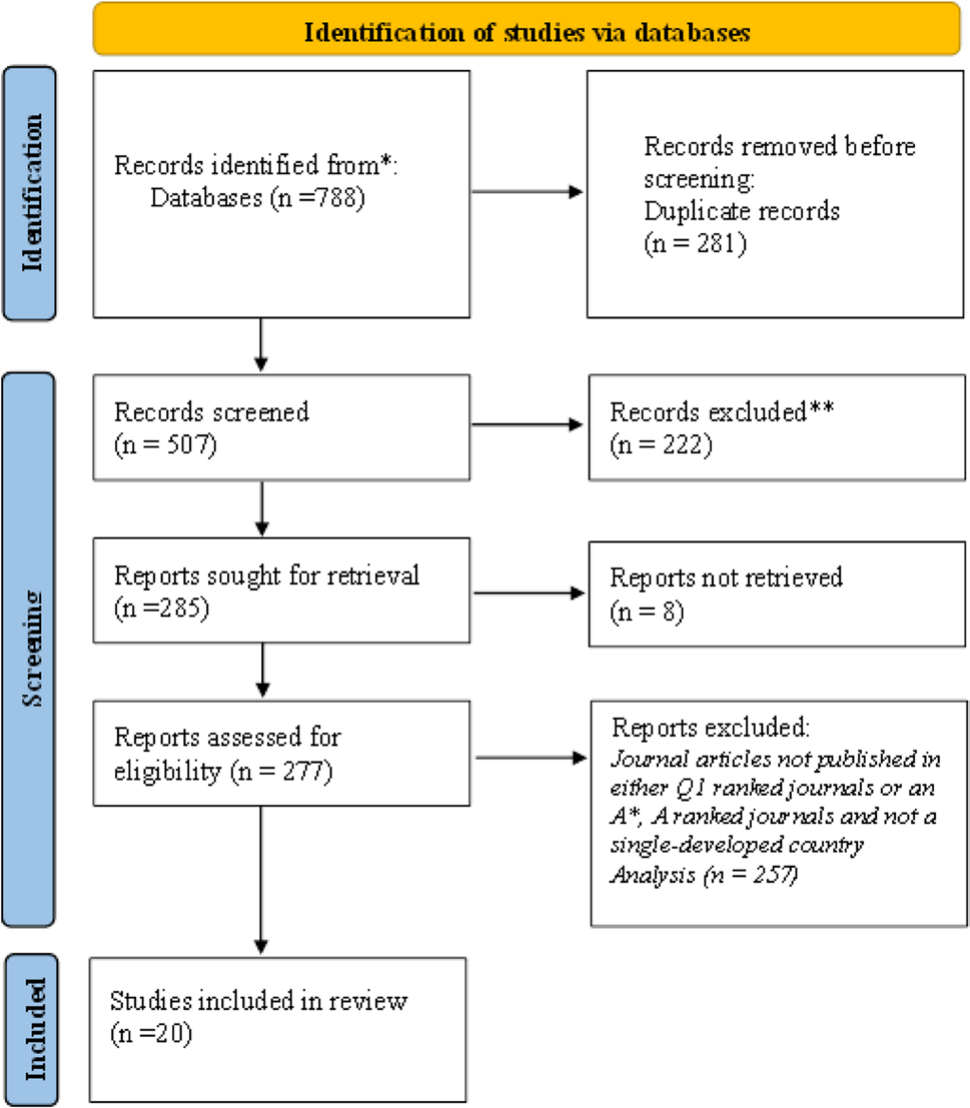
PRISMA flowchart for the identification, screening, eligibility, and studies included in SLR

To begin with the review, we have collected a total of 788 search results from the aforementioned databases. Subsequently, we traced 281 duplicates in the search results and eliminated those records prior to commencing the record screens. Then, the remaining 507 articles underwent a screening process based on the abstract content and 285 articles were selected to further assessment, and the remaining articles were excluded from the study due to the unsuitable content. During the following phase, the articles underwent a rigorous full-text screening and a total of 277 articles were selected for eligibility assessment. However, during this stage, eight articles were excluded due to the restricted access to those full-text articles. Figure 1 presents the sequential steps involved in identifying the related articles for the SLR via databases, as depicted in the PRISMA flowchart.

### Articles inclusion and exclusion criteria

In this phase, the 277 selected full-texts were reviewed sensibly in accordance with the predefined article inclusion and exclusion criteria described in Table 2. An in-depth assessment was conducted to determine whether each article met the predefined criteria for inclusion in the study. In adherence to the inclusion and exclusion criteria, we selected 20 articles from developed contexts for this SLR. Moreover, the Human Development Index (HDI)^1^ is utilized as the criteria for distinguishing developed countries in this SLR.

**Table 2:** Inclusion and exclusion criteria

	Inclusion criteria	Exclusion criteria
1.	The article should be published in a journal with Q1 or an A* or A ranked journal and written in English. If an article is ranked in both the Scimago Journal Ranking (SJR) and the Australian Business Deans Council (ABDC) list, it should be a Q1 ranking in SJR and an A* or A ranking in ABDC list, respectively.	Papers that are published in journals with a ranking below Q1 or A and written in other than English. If an article is ranked in both the Scimago Journal Ranking (SJR) and the Australian Business Deans Council (ABDC) list, it is not a Q1 ranking in SJR and an A* or A ranking in ABDC list, respectively.
2.	Should be focused on the context of developed countries.	Papers that focused on the context of developing countries
3.	Should be a single-country analysis	Papers that focused on multi-country analysis and not primarily focus on single-country analysis

### Data extraction and summarization

Then, we extracted all required information from the 20 papers that were eligible for this SLR according to the eligibility criterion. All the selected papers reviewed, extracted, and summarized within an Excel sheet in terms of different themes including author(s) name, published year, article title, the title of the publication, objectives, study period, context, proxy variables, estimation method, and key findings to identify the heterogeneity across the studies.

### Quality assessment

As a crucial phase of SLR, we ensure the quality of articles included in our SLR. Thus, an assessment was conducted to determine the level of quality of all 20 papers that met the eligibility criteria for inclusion in this SLR via utilizing the checklist of Al-Emran et al. (2018). The Al-Emran et al. (2018) checklist reviews each paper to ascertain whether they encompass clear and complete research objectives, explicit proxies and methodology, estimation procedures, and present results according to expected scientific standards. The quality assessment checklist and quality assessment results are presented in Appendices 1 and 2, respectively. The quality levels of all selected papers included in this SLR were recorded above 75%.

## Study sample credentials

Before analyzing the major characteristics of examined papers, the researchers observed the general credentials of the study sample of this SLR. At the very outset, “Distribution of the studies by country” and “Distribution of the studies by year of publication and journal of publication” provide an overview of the examined papers, basically their distribution across the developed countries, the year of publication, and the journal of publication.

### Distribution of the studies by country

**Fig. 2:**
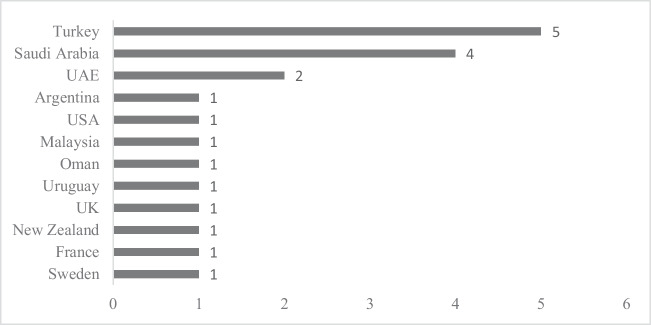
Distribution of the studies in terms of country

As per the inclusion criteria, all selected articles for the analysis were limited to single-country analysis in the developed country setting because the single-country analysis would offer a more focused and concise representation of the relationship between financial development and environmental quality rather than a multiple-country analysis. Figure 2 visually presents the distribution of selected studies across the developed countries where these studies were carried out. Notably, 25% of the selected articles were carried out in Turkey, and 20% of the studies were from Saudi Arabia. However, Argentina, the USA, Malaysia, Oman, Uruguay, the UK, New Zealand, France, and Sweden accounted for a combined 45% contribution to the study sample, while a 10% contribution was from the United Arab Emirates (UAE). Furthermore, 65% of the selected articles were from the Asian context.

### Distribution of the studies by year of publication and journal of publication

Twenty analyzed articles sourced from high-quality (Q1 or A or A*) journals were published over the past decade. Among these articles, the majority are from Environmental Science and Pollution Research (10 articles) and the remaining articles are from the following journals: Renewable and Sustainable energy Review (2 articles), Sustainability (2 articles), Energy Economics (2 articles), Frontiers in Environmental Science (2 articles), Economic Modelling (1 article), and Technological Forecasting and Social Change (1 article). In addition, it is important to note that all the analyzed articles were published during the period of 2013–2022. Among these articles, 2 articles were published in 2013 while one article was published in 2016 and 2017 each year. Moreover, the dispersal of selected studies across the succeeding years was 3 articles in 2018, 5 articles in 2020, 4 articles in 2021, and another 4 articles in 2022.

## Results

This section primarily focuses on discussing the key characteristics and major findings of the examined papers in this SLR.

### Major Study Characteristics

We extracted major attributes of the examined papers to recognize the heterogeneity of study characteristics and outcomes. Firstly, it was observed that scholars employed diverse time intervals to explore the link between financial development and environmental quality within developed country settings. Importantly, 90% (18 papers) of articles employed annual data to account for the linkage between study variables, and the remaining articles (Alam et al. 2022; Shahbaz et al. 2020) utilized quarterly data to enhance the study aims.

Secondly, all studies included in this study used quantitative techniques to estimate the association among the study variables. All the studies in this study sample undertook to estimate the long-run dynamics and 17 articles out of the sample, employed short-run analysis to measure and explore the short-run dynamics pertaining to the study context. Regarding the estimation techniques used in the examined articles, we observed that Autoregressive Distributed Lag (ARDL) approach is a widely used econometric model (85%) with numerous variants of it. These variants include ARDL bound test approach in 11 articles, Fourier ARDL (Serener et al. 2022), Bootstrap ARDL (Shahbaz et al. 2018, 2020a), Non-linear ARDL (Raggad 2020), Quantile Autoregressive Distributed Lag model (Godil et al. 2020), and Dynamic ARDL Simulations Approach (Khan et al. 2021). Interestingly, in addition to the ARDL approach the examined articles employed a diverse range of regression types as alternative estimation techniques. The stochastic impacts by regression on population, affluence, and technology (STIRPAT model), Dynamic OLS, and Fully Modified OLS are adapted alongside the ARDL estimations. Furthermore, 75% of examined articles in this study incorporated various causality tests as an integral part of the econometric analysis, and details of econometrics models applied by the examined papers are given in Table 3.

**Table 3:** Econometrics estimations of eligible studies

Authors	Econometric tool
Charfeddine and Khediri (2016)	Gregory and Hansen approach Hatemi-J approach Vector Error Correction Model (VECM) causality analysis
Kahouli et al. (2021)	Autoregressive Distributed Lag Model (ARDL) Vector Error Correction Model (VECM) causality analysis
Serener et al. (2022)	Fourier Autoregressive Distributed Lag Model Fourier Toda Yamamoto (Fourier TY) causality test
Shahbaz et al. (2018)	Bootstrap Autoregressive Distributed Lag Model Granger causality based on bootstrapping ARDL
Shahbaz et al. (2020)	Gregory-Hansen cointegration test Toda-Yamamoto causality test
Aljadani (2022)	STIRPAT model with Autoregressive Distributed Lag Model Pairwise Granger causality tests
Xu et al. (2018)	Autoregressive Distributed Lag Model (ARDL) Vector Error Correction Model (VECM) causality analysis
Villanthenkodath and Arakkal (2020)	Autoregressive Distributed Lag Model (ARDL)
Shahbaz et al. (2020a)	Bootstrap Autoregressive Distributed Lag Model Variance Decomposition Analysis and Impulse Response
Raggad (2020)	Nonlinear Autoregressive Distributed Lag Model (NARDL) Asymmetric causality test of Hatemi-J
Awosusi et al. (2022)	Autoregressive Distributed Lag Model (ARDL) Frequency Domain Causality Test
Alam et al. (2022)	Autoregressive Distributed Lag (ARDL) Dynamic Ordinary Least Square
Shahbaz et al. (2013)	Autoregressive Distributed Lag Model (ARDL) Vector Error Correction Model (VECM) causality analysis
Godil et al. (2020)	Quantile Autoregressive Distributed Lag model
Khan et al. (2021)	Dynamic Autoregressive Distributed Lag Model Simulations Approach
Rjoub et al. (2021)	ARDL bounds test Bayer–Hanck cointegration test Fully-modified Ordinary Least Square (FMOLS) Dynamic Ordinary Least Square (DOLS) Canonical Cointegrating Regression (CCR)
Adebayo et al. (2021)	Autoregressive Distributed Lag Model (ARDL) Fully-modified Ordinary Least Square (FMOLS) Dynamic Ordinary Least Square (DOLS) Toda-Yamamoto causality test
Cetin et al. (2018)	Autoregressive Distributed Lag Model (ARDL) Vector Error Correction Model (VECM) causality analysis
Ozturk and Acaravci (2013)	Autoregressive distributed lag Model Granger causality test
Katircioğlu and Taşpinar (2017)	Dynamic Ordinary Least Square (DOLS) Granger causality Test

Thirdly, we analyzed the major proxy variables employed by the examined papers of this SLR. Using diverse econometrics approaches, eligible papers explored the link between financial development and environmental quality. Within this context, we observed the proxy variables employed by the authors under three categories, namely (1) proxy variables for financial development, (2) proxy variables for environmental quality, and (3) other variables employed in econometric estimations.

**Fig. 3:**
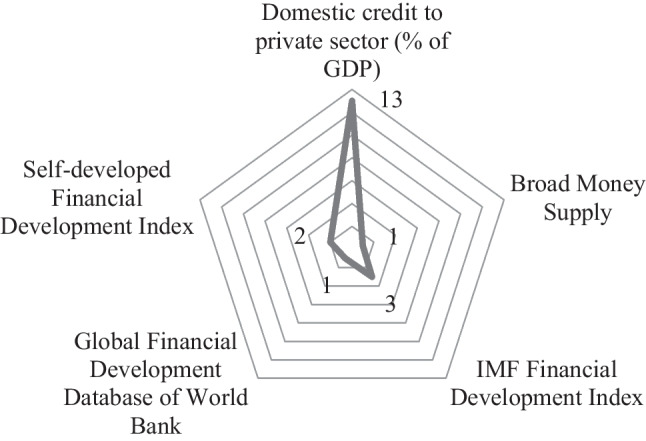
Distribution of proxies employed in eligible studies

Under the first category, several proxy variables were employed to assess and quantify the diverse aspects of financial development. Figure 3 depicts a graphical representation of proxies used to measure financial development in the examined articles in this review. Mainly, the proxy most commonly used by researchers, included in 13 articles, is domestic credit to the private sector (% of GDP) which captures the bank-based financial depth improvement. Similarly, another bank-based financial depth improvement proxy, the broad money supply is used by Shahbaz et al. (2020a). Two studies measured financial development via a self-developed index by principal component analysis (Katircioğlu and Taşpinar 2017; Shahbaz et al. 2020). However, Shahbaz et al. (2020) accounted financial depth and efficiency of both bank-based and market-based development by incorporating different variables, while Katircioğlu and Taşpinar (2017) focused solely on bank-based financial depth and efficiency. The remaining studies used the financial development index of the International Monetary Fund (Adebayo et al. 2021; Awosusi et al. 2022), measured financial depth, access, and efficiency of financial markets and intermediaries; global financial development database of World Bank (Godil et al. 2020) accounted financial stability along with financial depth, access and efficiency of financial markets and intermediaries.

In the second category, the proxies of environmental quality across all the studies examined in this SLR primarily relied on two proxies. We observed that the predominant proxy employed for measuring the environmental quality was carbon dioxide (CO_2_) emission and it was employed by 18 articles. Conversely, the remaining two studies conducted by Godil et al. (2020) and Alam et al. (2022) preferred carbon footprint for measuring environmental quality.

**Fig. 4:**
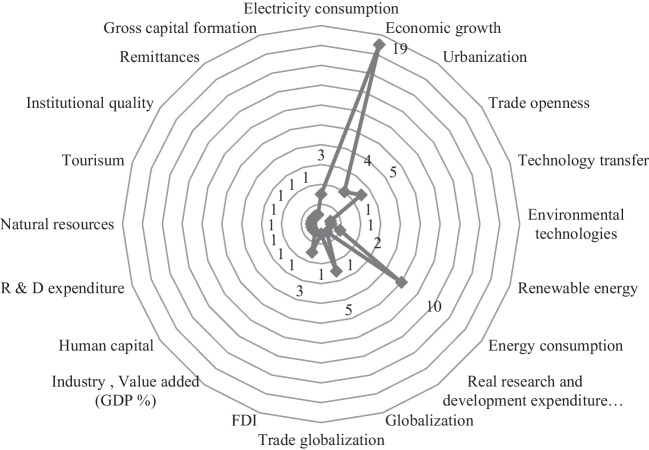
Distribution of other variables employed in eligible studies

Next, other variables used in econometric models in 20 papers included in this SLR are presented in Figure 4. Generally, the inclusion of economic growth as a vital proxy within econometric models has been widely acknowledged in the majority of articles, as evidenced by the examination of 19 articles, and these studies highlight the significance of measuring economic growth, as it plays a key role in inspecting the theoretical relationship between economic progress and environmental quality. However, Kahouli et al. (2021) adopted a different approach by excluding economic growth from the econometric model. In addition, articles employed a varying number of variables in accordance with the study context.

### Theory tested/used

Grossman and Krueger (1991) observed an inverted U-shaped relationship between economic growth and environmental deterioration, commonly known as the Environmental Kuznets Curve (EKC). This concept was earlier proposed by Kuznets (1955) concerning the inverted U-shaped relationship between economic growth and income inequality. By adopting this conceptualization of EKC, a total of 10 articles in this study examined the existence of the EKC phenomenon and demonstrated the presence of EKC in the study context (Alam et al. 2022; Cetin et al. 2018; Charfeddine and Khediri 2016; Godil et al. 2020; Katircioğlu and Taşpinar 2017; Khan et al. 2021; Ozturk and Acaravci 2013; Shahbaz et al. 2018, 2020a; Villanthenkodath and Arakkal 2020).

Besides, empirical investigations have been undertaken to understand the interconnection between financial development and environmental outcomes. Those studies have adopted the EKC framework as a conceptual framework to discover this relationship. By employing the squared term of financial development proxy, the studies aimed to capture the non-linear relationship between financial development and environmental quality. In total, five studies included in this SLR comprehensively investigated the inverted U-shaped relationship between financial development and environmental quality (Charfeddine and Khediri 2016; Shahbaz et al. 2013, 2018, 2020, 2020a) and utilized the squared term of financial development proxy. Among these studies, all the studies established the presence of an inverted U-shaped relationship between financial development and environmental quality, except Shahbaz et al. (2013) in the Malaysian economy. These scholarly works provided empirical confirmation supporting the indication that as in financial advancement in developed economies, there is an early phase in which environmental quality worsens, followed by an eventual improvement as financial development reaches a peak level. In addition, Shahbaz et al. (2020) extended the relationship between financial development and environmental quality beyond the conventional inverted U-shaped, and the polygonal relationship between financial development and environmental quality was tested and confirmed the inverted N-shaped relationship between financial development and environmental quality.

### Summary of key findings

In this section, we attempt to summarize the key findings of the 20 papers included in this study. Mainly, the key findings can be generally categorized into four main groups, providing comprehensive insights into the research landscape. Table 4 presents the relationship between financial development and environmental quality, as empirically demonstrated by the studies included in this SLR.

**Table 4:** Key findings of the studies included in the SLR

#	Study	Positive relationship	Negative relationship	No relationship	Mix results
1.	Charfeddine and Khediri (2016)		√		
2.	Kahouli et al. (2021)		√		
3.	Serener et al. (2022)		√		
4.	Shahbaz et al. (2018)	√			
5.	Shahbaz et al. (2020)		√		
6.	Aljadani (2022)	√			
7.	Xu et al. (2018)		√		
8.	Villanthenkodath and Arakkal (2020)	√			
9.	Shahbaz et al. (2020a)		√		
10.	Raggad (2020)				√
11.	Awosusi et al. (2022)			√	
12.	Alam et al. (2022)	√			
13.	Shahbaz et al. (2013)	√			
14.	Godil et al. (2020)		√		
15.	Khan et al. (2021)		√		
16.	Rjoub et al. (2021)		√		
17.	Adebayo et al. (2021)		√		
18.	Cetin et al. (2018)		√		
19.	Ozturk and Acaravci (2013)			√	
20.	Katircioğlu and Taşpinar (2017				√

The first set of studies (5 studies) supports in establishing a positive relationship between financial development and environmental quality in developed countries. The findings of these studies indicate that advancement in financial development in developed countries tends to improve the environmental quality. The studies of Alam et al. (2022), Shahbaz et al. (2013, 2018), and Villanthenkodath and Arakkal (2020) show that financial development improves environmental quality by reducing carbon emission in France, New Zealand, Oman, and Malaysia, respectively, both in the long run and the short run. Similar results have been established in the Saudi Arabian economy by a recent study by Aljadani (2022) which further analyzed the uncovered dimension of the technological effect on financial development. However, the findings of Aljadani (2022) challenge the previously established notion of a positive effect of financial development and suggest that the technology associated with financial development may actually have a detrimental impact on environmental quality in Saudi Arabia.

The second set of studies contributes to confirming a negative relationship between financial progress and environmental quality in developed economies. Majority of the studies (11 studies) included in this SLR provide evidence to conclude that financial development is a crucial determinant of environmental degradation in developed economies (Adebayo et al. 2021; Cetin et al. 2018; Charfeddine and Khediri 2016; Godil et al. 2020; Kahouli et al. 2021; Khan et al. 2021; Rjoub et al. 2021; Serener et al. 2022; Shahbaz et al. 2020, 2020a; Xu et al. 2018)

The next set of studies (2 papers) provides evidence to establish an insignificant relationship between financial development and environmental quality in developed economies. Those empirical analyses did not find a significant relationship between financial development and environmental quality in developed economies. The studies conducted by Awosusi et al. (2022) and Ozturk and Acaravci (2013) verified an insignificant relationship between financial development proxies and environmental quality in Saudi Arabia and Turkey, respectively.

The final set of studies (2 articles) emphasizes mixed shreds of evidence to the body of literature on the relationship between financial development and environmental quality in developed country settings. Raggad (2020) investigated Saudi Arabia, and Katircioğlu and Taşpinar (2017) examined the Turkish economy and established both positive and negative effects of financial development on the environment. As per Raggad’s (2020) findings, in the long run, negative shocks in financial development surge carbon emissions, and positive shocks in financial development have shown an improvement in environmental quality in the short run. Katircioğlu and Taşpinar (2017) highlighted that financial development plays a moderating part in the relationship between real output and carbon emissions, with different effects observed in the long run and short run. The study findings imply that as real output increases, the carbon emissions are mitigated through financial development in the short run while in the long run financial development moderates the effect of real output on carbon emissions positively.

Very importantly, significant variations in the findings were observed within the Saudi Arabian context. Besides, authors employed different variants of the ARDL approach and diverse study periods to enhance the objectives of their respective studies (Aljadani 2022; Kahouli et al. 2021; Raggad 2020; Xu et al. 2018). Additionally, a similar pattern was observed within the Turkish context with diverse econometric models and time frames in their studies (Cetin et al. 2018; Godil et al. 2020; Katircioğlu and Taşpinar 2017; Ozturk and Acaravci 2013; Rjoub et al. 2021).

## Discussion

The objective of this SLR is to synthesize the existing knowledge and wisdom on the relationship between financial development and environmental quality in developed countries. In this SLR, we covered a total of 20 articles to discover whether financial development significantly influences environmental quality in developed countries. Answering the research question, it was found that 90% of studies analyzed in this SLR concluded that financial development significantly influenced (either improve or worsen) the environmental quality in developed economies. The first aspect explored regarding the relationship between financial development and environmental quality revealed that approximately 60% of study findings marked that progress in the financial industry deteriorates the ecological quality in developed countries. The second aspect concerns financial development enhances environmental quality by reducing carbon emissions in developed economies. Additionally, the third aspect highlights financial development which can have a varying impact (improve or worsen) in different time periods such as long-run and short-run, and in total 40% of findings support establishing the second and third aspects.

### Knowledge gaps and way forward for future research

Financial development comprises various dimensions that jointly contribute to the functioning and effectiveness of a country’s financial system. These dimensions include financial access, financial depth, financial efficiency, and financial stability (Čihák et al. 2012; Mollaahmetoğlu and Akçalı 2019). Financial access refers to the extent to which individuals and firms can use and access financial markets and institutions to meet their financial requirements (Čihák et al. 2012). The size and breadth of financial markets and institutions within an economy, including the wide range of financial products and services available is known as financial depth (Čihák et al. 2012). Financial efficiency refers to the effectiveness and productivity of financial markets and institutions that provide financial services, including the speed and cost-effectiveness of transactions (Čihák et al. 2012). Financial stability refers to the soundness and resilience of the financial system, ensuring that it can absorb shocks and disruptions without compromising the efficient operation of financial intermediation, financial markets, and the overall economy (Čihák et al. 2012). Therefore, when modeling the impact of financial development on environmental quality in developed countries, it is imperative to consider and incorporate all these dimensions of financial development. Specifically, there is a lack of studies that discover all aspects of financial development, including financial depth, financial efficiency, financial access, and financial stability. While some studies concentrated on specific dimensions such as the development of financial markets including the stock market and credit market, bank-based development and others explored different aspects.

**Diagram 1:**
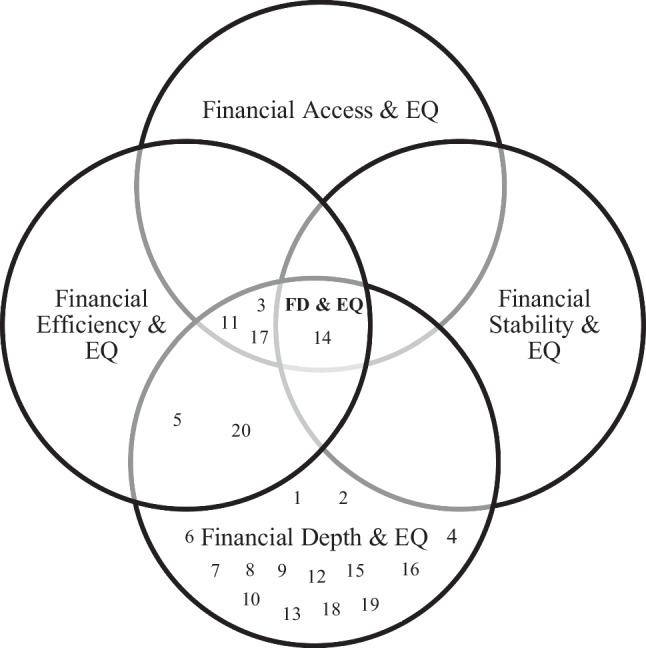
Analysis of examined papers. FD and EQ stands for Financial Development and Environmental Quality, respectively. 1. Charfeddine and Khediri (2016) 2. Kahouli et al. (2021) 3. Serener et al. (2022) 4. Shahbaz et al. (2018) 5. Shahbaz et al. (2020) 6. Aljadani (2022) 7. Xu et al. (2018) 8. Villanthenkodath and Arakkal (2020) 9. Shahbaz et al. (2020a) 10. Raggad (2020) 11. Awosusi et al. (2022) 12. Alam et al. (2022) 13. Shahbaz et al. (2013) 14. Godil et al. (2020) 15. Khan et al. (2021) 16. Rjoub et al. (2021) 17. Adebayo et al. (2021) 18 Cetin et al. (2018) 19. Ozturk and Acaravci (2013) 20. Katircioğlu and Taşpinar

The following Diagram 1 presents the analysis of examined empirical studies of this SLR using a Venn diagram. The examination of interactions among the four strands of literature enables a clear identification of the relationships and interconnectedness between them. Interestingly, 70% of studies have employed one of the significant dimensions of bank-based development, domestic credit to the private sector as the proxy for capturing financial development. It explains the depth of financial institutions only. Two studies (10% of studied articles) have taken a comprehensive approach by capturing financial development through the proxies of both financial depth and financial efficiency. Fifteen percent of studied articles have recognized financial depth, efficiency, and access as proxy variables of financial development to explore its effect on environmental quality in different developed countries. More importantly, 5% of examined empirical studies in this SLR used all dimensions to capture the financial development and check its impact on environmental quality. A notable observation in the standing literature is the lack of scholarly works that precisely focused on all dimensions of financial development to explore its impact on environmental quality in developed country settings. As such, the prevailing conclusions regarding the impact of financial development on environmental quality cannot be generalized to developed contexts because existing knowledge fails to accurately capture how financial development truly affects environmental quality in developed countries. Thus, it will be significant to bridge this knowledge gap and straddles all four dimensions of financial development to explain their collective impact on environmental quality.

The escalation in greenhouse gas concentrations is primarily responsible for the warming of the atmosphere (Zubair et al. 2023). In addition to carbon dioxide, nitrous oxide (N_2_O), sulfur dioxide (SO_2_), methane (CH_4_), ozone (O_3_), and fluorinated gases (F-gases) including hydrofluorocarbons (HFCs), sulfur hexafluoride (SF6), and chlorofluorocarbon (CFC) are recognized as parts of greenhouse gas (Zubair et al. 2023). However, there is an absence of comprehensive studies that consider the holistic nature of environmental quality. Eighty percent of the examined articles in this SLR utilized carbon dioxide (CO_2_) as the proxy for environmental quality. As such, this gap in the existing body of literature emphasizes the requirement for further inquiry that discovers the effect of financial development by encompassing all greenhouse gases rather than exclusively focusing on carbon dioxide emissions.

### Contribution of the study

This study incrementally contributes to the existing literature by identifying the current understanding of the effect of financial development on environmental quality in developed economies. Through this SLR, we have been able to discuss the existing empirical evidence on the nexus between financial development and environmental quality in developed nations. The findings have implied that financial development significantly impacts environmental quality, either improving or worsening the environmental quality in developed economies which is a helpful guide to economists and policymakers to set the policies to enhance sustainable financing practices in developed economies. In addition, no compelling SLR is known to discuss the existing knowledge gaps in this study domain to create a pathway for future scholars, and this study guides future researchers to take a way forward in this research domain.

### Limitations

This SLR extracted papers from an extensive search across the key databases of Web of Science, Scopus, and Google Scholar. In addition, this study included articles published in Q1 or an A, A* journals to ensure the high quality of reviewed articles. However, we excluded other outlets such as working papers and lower-ranked articles, even though those may be potentially relevant to the current review. This study primarily reviewed the single-country studies to get a precise understanding of the relationship between financial development and environmental quality in developed countries by omitting the multi-country studies. Thus, future scholars can benefit from including working papers, other ranked journals, and multi-country studies which would add value to the existing body of knowledge.

## Conclusion

This SLR investigated the existing body of knowledge relating to the impact of financial development on environmental quality in developed economies. This review analyzed a total of 20 high-quality articles out of the initial selection of 277 studies published in seven journals over the last decade. All the examined articles are single-country studies and are spread around 12 developed countries across different regions. This SLR used the recommended guideline of the PRISMA statement in conducting the study. The studies varied in terms of study periods and selected variables for measuring financial development, environmental quality, and other variables. However, most of the analysis is based on the ARDL approach and its variants. Overall, the analysis result shows that financial development significantly enhances or degrades the environmental quality in developed economies. In addition, a substantial knowledge gap is visible in this research domain that highlights further studies which capture the financial development and environmental quality link from different proxies.

## Data Availability

The complete information sheet used for the study may be supplied by the corresponding author on reasonable request.

## Appendix

See Tables 5 and 6

**Table 5:** Quality assessment checklist

#	Question
1.	Are the research aims clearly specified?
2.	Was the study designed to achieve these aims?
3.	Are the variables considered by the study clearly specified?
4.	Is the study context/discipline clearly specified?
5.	Are the data collection methods adequately detailed?
6.	Does the study explain the reliability/validity of the measures?
7.	Are the statistical techniques used to analyze the data adequately described?
8.	Do the results add to the literature?
9.	Does the study add to your knowledge or understanding?

Adapted from Al-emran et al. (2018)

**Table 6:** Quality assessment results

Study	Q1	Q2	Q3	Q4	Q5	Q6	Q7	Q8	Q9	Total	%
Charfeddine and Khediri (2016)	1	1	0.5	1	0.5	1	1	0.5	1	7.5	83%
Kahouli et al. (2021)	1	1	0.5	1	1	1	1	1	1	8.5	94%
Serener et al. (2022)	0.5	1	1	1	0.5	1	1	1	1	8	89%
Shahbaz et al. (2018)	0.5	1	0.5	1	1	1	1	1	1	8	89%
Shahbaz et al. (2020)	0.5	1	1	1	1	1	1	1	0.5	8	89%
Aljadani (2022)	0.5	1	0.5	1	1	0.5	1	1	0.5	7	78%
Xu et al. (2018)	1	1	1	0.5	1	1	1	0.5	0.5	7.5	83%
Villanthenkodath and Arakkal (2020)	1	1	1	1	0.5	1	1	1	1	8.5	94%
Shahbaz et al. (2020a)	0.5	1	1	1	1	1	1	1	1	8.5	94%
Raggad (2020)	1	1	1	1	1	1	0.5	1	0.5	8	89%
Awosusi et al. (2022)	1	1	1	1	0.5	1	1	1	1	8.5	94%
Alam et al. (2022)	0.5	1	1	1	1	1	1	1	1	8.5	94%
Shahbaz et al. (2013)	1	1	1	1	1	1	1	1	0.5	8.5	94%
Godil et al. (2020)	1	1	0.5	1	0.5	1	1	1	1	8	89%
Khan et al. (2021)	1	1	1	1	0.5	1	1	1	0.5	8	89%
Rjoub et al. (2021)	1	0.5	1	1	1	0.5	1	0.5	0.5	7	78%
Adebayo et al. (2021)	0.5	1	1	1	1	1	1	1	1	8.5	94%
Cetin et al. (2018)	1	1	1	0.5	1	1	1	1	1	8.5	94%
Ozturk and Acaravci (2013)	1	1	1	1	1	1	0.5	0.5	1	8	89%
Katircioğlu and Taşpinar (2017)	1	1	0.5	1	1	1	1	1	0.5	8	89%

The scoring procedure involved allocating points to each question based on a three-point scale: a response of “Yes” was given a value of 1, a response of “No” was assigned a value of 0, and a response of “Partially” was assigned a value of 0.5

## Footnotes

1 The Human Development Index (HDI) is a statistical tool utilized to gauge a country’s comprehensive progress in both its social and economic aspects.
